# A qualitative study of referring agents’ perceptions of access barriers to inpatient substance abuse treatment centres in the Western Cape

**DOI:** 10.1186/s12954-015-0064-z

**Published:** 2015-09-26

**Authors:** Deborah Isobell, Kamal Kamaloodien, Shazly Savahl

**Affiliations:** Psychology Department, University of the Western Cape, Robert Sobukwe Road, Cape Town, South Africa

**Keywords:** Substance abuse, Perceptions, Treatment, Barriers to treatment, Access, Inpatient treatment, Substance abuse treatment centres, Referral system, Referral agents

## Abstract

**Background:**

Despite empirical support for the individual and public health benefits of treating substance use disorders (SUDs) , access to these services is impeded by several barriers. Although many studies on access barriers have been put forward in the literature, few have explored the barriers to accessing state-funded inpatient substance abuse treatment or the views of referral agents.

**Methods:**

A qualitative study was conducted to explore referring agents’ perceptions of the barriers to accessing state-funded inpatient substance abuse treatment centres in the Western Cape Province of South Africa. Six individual in-depth interviews were conducted and analysed using theoretical thematic analysis.

**Results:**

The key barriers to emerge from the analysis pertained to referring agents’ perceptions of the following: service users, the substance abuse referral and treatment system and community dynamics.

**Conclusions:**

Recommendations are made for interventions to address the identified barriers.

## Background

Substance use is one of the major public health concerns in South Africa, with the Western Cape Province particularly affected [1]. Access to facilities providing treatment services is integral to addressing substance use disorders (SUDs) and its related problems [2, 3]. Yet despite empirical support for the individual and public health benefits of treating SUDs [4], access to these facilities is impeded by several barriers which include the affordability and availability of treatment services [5] and stigma [6].

Inpatient treatment is indicated for treatment seekers who “need a stable and safe living environment…have more severe addiction and comorbidities…and may be at high risk of relapse, mental health crisis or behavioral problems” ([7], p.119). In facilitating service users’ access to relevant and holistic treatment services, referrals are essential to ensuring the continuum of care [8]. However, referrals also carry the risk of “losing" the service user [9] if there are barriers. Thus, effective referral systems lead to more cost effective, sustainable treatment services as it regulates the availability of and appropriate provision of care [10]. The South African Community Epidemiology Network on Substance Abuse [1] has identified various sources of referrals to treatment services, of which the social services/welfare sector is an important source. Thus, ascertaining the perceptions of these referral agents within this sector is important in identifying barriers to treatment accessibility.

Although many studies on access barriers have been put forward in the literature [2, 5, 11, 12], there is a paucity of literature on the barriers to accessing state-funded inpatient substance abuse treatment [13] or the views of referral agents. Access to treatment, for the purpose of this study, refers to the ability to link to state-funded treatment facilities from a lower level of care.

As revealed in a South African access study on the barriers to inpatient and outpatient treatment, [2] treatment system related factors that have a bearing on access to treatment include the following:

- The absence of a structured referral pathway
- The need for formal referrals from a social worker
- Long referral processes
- Undergoing mandatory detoxification or mental health treatment services prior to admissions to treatment facilities as these services are not integrated
- Service user eligibility criterion and
- Extensive waiting time

The primary aim of this study was to explore the access barriers to inpatient substance abuse treatment facilities from the perspective of referring agents in the Western Cape. This information will contribute to the dialogue on the barriers to inpatient treatment services and could potentially enhance service provision.

Bronfenbrenner’s Process-Person-Context-Time (PPCT) model was used to theoretically ground the study. According to the model, individuals wield influence on their surrounding environments, which in turn influence them [14]. Considering that barriers to treatment occur at different levels of interaction (i.e. structural and non**-**structural [6] or internal and external [12]), this model provides a lens to locate and understand the factors that impact upon treatment access, holistically.

## Methods

Considering the aim and the exploratory nature of the study, a qualitative methodological framework was utilised. Participants were recruited from NGOs and government facilities to ultimately include six referring agents from two community clinics and four outpatient centres rendering substance abuse treatment services. Participants were required to be actively employed in the substance abuse treatment system, aid referrals to inpatient treatment facilities, hold recognised qualifications and belong to a professional body at the time of the study to be eligible for inclusion. Of the registered counsellor, psychologist and four social workers who participated in the study, five were female. Professional experience across the sample varied between 1 and 10 years.

Data was collected by means of semi-structured in-depth interviews and guided by an interview schedule (Appendix A). Questions centred on the substance abuse treatment system, the process of accessing state-funded inpatient treatment, perceptions of barriers and enablers of access and referring agents’ views on treating substance abuse. Informed consent was obtained prior to the commencement of interviews, which were conducted at participants’ place of work and lasted between 30**–**90 min. Each interview was audio recorded.

Audio recordings of interviews were transcribed verbatim and analysed using Braun and Clarke’s [15] six steps of thematic analysis. To become familiar with the data, interviews were transcribed by the first author, and initial codes were assigned to the data. With conceptual input from the second and third author, themes were identified and evaluated. Finally, themes were described and named.

Ethical clearance was obtained from the University of the Western Cape.

## Results

The salient findings to emerge from the analysis broadly pertained to referring agents’ perceptions of service users, the substance abuse treatment and referral system and its practices and community dynamics.

### *Person*-related barriers

#### Motivation for treatment

Referring agents emphasised the critical role of motivation for treatment in processes of accessing care. Engaging in sessions at an outpatient treatment centre was regarded as the necessary first step for building motivation. Some participants preferred that service users verbalised their need for inpatient treatment to demonstrate their motivation for treatment, while for others, a lack of motivation directly prohibited a referral:

“…we need to establish that they are ready to change, motivated to change, and to maintain that change. In order to do that they need to belong to the programme … and that is what we do, we would want them to be part of our programme and then I would want, or it would be best for the person if they elicit a response where they say, I would rather want to go to inpatient.” (Participant 1)

“…if the person is not really motivated and then does not want to go to an inpatient centre, so I would not be able to send that particular person…” (Participant 6)

Motivation was said to be so critical that even in the context of an “ideal” environment for treatment, with accessible inpatient treatment services, finances and support for treatment, these factors proved irrelevant in its absence. Alternatively, an individual may be motivated for treatment but, faced with several barriers, be unable to access treatment services:

“Maybe if they’re not motivated, maybe they have all the resources, they have access, their family is able to pay…they have access to NA, they have access to their religion, religious community is able to support them, then that person is not ready for treatment, ok. Maybe the person IS ready for treatment and then they don’t have access… they don’t have access to the treatment centre and it’s too far.” (Participant 1)

While admission to treatment centres became increasingly unlikely due to waning motivation, delays in entry to treatment had the potential to end fatally:

“…motivation is the biggest thing,…motivation is the biggest thing that I find with the client, that happens because for the addict, most probably the most important thing in his life is his addiction, so while his in that space, that’s all he focuses on, there’s nothing more important for him, but when it comes to that point of change, you know, there’s that small space where that person is wanting space and if you don’t take it, wanting change and if you don’t take advantage of that, you know, that time he falls back. It’s difficult to get him back to that point of wanting help again.” (Participant 2)

“…with substance abuse, you sort of need to motivate that person now and if they say yes, you’ve got to grab it right there because circumstances change and if you wait too long or wait 6 months the patient’s either died or they have lost interest or are in a different space and are not motivated to go anymore…” (Participant 5)

Though factors related to the treatment system may prohibit service users from following through and entering treatment facilities, fluctuating motivation also had a bearing on the decision to pursue treatment, one referring agent remarked:

“Motivation changes continually, so it’s not always the treatment facility’s fault that the client didn’t follow through. It could be the space where he is at in his motivation.” (Participant 2)

### Stigma towards women

Participants believed that women faced added barriers, such as stigma, and were often turned away from treatment due to being pregnant or missed opportunities to slot into treatment due to childcare needs:

“…for women especially, they don’t want to come. They don’t want to come to a place like this because as a woman, what are you doing in the first place using?” (Participant 1)

“…because of their status, they do not have responsibilities like children, or they don’t want to be judged and most females that are entering our programme, they are referrals from social development. Children have been removed out of their care, etcetera etcetera. That’s the only type of females that we get here. Other than that it makes you wonder what are the other females doing. Why don’t they access our services? Other than that our other females will be referrals from school, like learners.” (Participant 4)

Often, only when children had been removed by authorities, would women seek help.

### Pregnancy

Pregnancy was also believed to hinder access to state-funded inpatient treatment facilities:

“…the ones that’s pregnant, it’s very difficult, depending on where they are at in their pregnancy and what they are on…a lot of them come here, they want to go…and they’re 8 months, on heroin, or Tik (Crystal Methamphetamine), and I cannot get them in…They usually drop out because there’s no open door, where do you go to?…the detox centre will take them but…where do they go to from there? There’s no rehab. Now outpatient is not gonna help them (at that stage) to stay clean and also they need to be monitored because of the baby and the dangers to the foetus, because what if she aborts…It’s very medically-complicated…It’s very difficult for pregnant ladies.” (Participant 1)

“…pregnant mothers, we can’t do that…inpatient facilities don’t allow that. They don’t have the resources. Some of them don’t even have you know at base nurses, so it is difficult it’s because of the safety of that person…it is policy and procedures as well, because they don’t have any facilities for pregnant mothers unless it’s a specialised inpatient facility…I’ve been working in Johannesburg, it’s very seldom they’ll approve an application where a mother is beyond three months. Sometimes when you get away with murder, when you’re still in the first trimester they will allow the person because the application was already approved, but anything thereafter they won’t.” (Participant 4)

Participants believed that the likelihood of access to treatment is impacted upon by service users' stage of pregnancy as well as capacity and resource issues at inpatient centres. Childcare considerations were also believed to impede access to inpatient treatment as mothers often had to forego opportunities to enter treatment on account of childcare needs.

### Childcare needs

“…If the person is a single parent and there’s no one looking after their children where do they put the children?” (Participant 1)

“…we often have a problem with is if a mother needs to go and has got kids, and there’s no- one to look after the kids then they can’t really go, or…then it becomes an issue of trying to place those kid during that time and then it’s a whole ‘nother system of the social workers going to court to try and find a placement for the two month…” (Participant 5)

Women appear to be required to evaluate what is more important: attaining sobriety or “fulfilling” their task as a mother.

### Disability

Disabilities were identified as another person-related barrier to accessing state-funded inpatient centres as the characteristics of treatment centres are not suited to different levels of physical functioning.

“Our physically disabled people aren’t being serviced at all, because the specifications of accessibility, they just don’t cater for that. I mean we struggle just getting a blind person in, then we struggle by getting a paraplegic in. They will consider the application, but I know for a fact they aren’t geared to service a disabled person. They don’t have the skill and neither do they have the resources.” (Participant 4)

Although this participant refers specifically to a past service users’ visual impairment constraining their engagement in a treatment programme, questions can also be raised around the degree of engagement that is possible for illiterate or partially literate substance users.

“…state (funded). They don’t even consider…We’ve never received any disabled client for that, physically disabled though. We only had one client…and it took us forever to get him in and eventually we got him in after a big fight, but…he didn’t make it because…they couldn’t…treat him, he couldn’t read the manual, he couldn’t participate effectively, the staff felt overwhelmed…” (Participant 4)

This referral agent also highlighted the specialised care needed by persons with disabilities, which facilities seemed to lack:

“…there’s certain beds that they need, certain care, it’s expensive. You can’t just ask any health (practitioner) or a nurse just…to be part of an inpatient facility and there’s a physically disabled person. We’re talking about bedsores, we’re talking about medication…some of them are diabetic, of which the inpatient facility didn’t prepare themselves for that. Now I know when you are an able bodied person and you are a diabetic, they still take you physically to your day hospital or the nearest hospital, you get your medication and they’ll bring you back. But it’s a different ball game, because the transport that they use aren’t accessible for that person…And I think it’s unfair to make a special facility just for disabled people, because that has nothing to do with them, for what we are fighting for and that is integration.” (Participant 4)

The importance of integrating substance abuse treatment services for persons with disabilities, whose needs appear to be unmet by state-funded facilities, was emphasised.

Also linked to physical well-being, active TB disease was identified as a person-related barrier to state-funded inpatient facilities.

### Tuberculosis

Active TB disease was expressed as prohibiting access to inpatient treatment services:

“Are there any clients you are not able to refer to treatment?” (Interviewer)

“Yes, some of my TB patients are a huge problem, because if they’ve been on treatment for two weeks or longer then it’s okay, but if…they’ve…defaulted on TB treatment it’s a huge issue because then we don’t’ know how resistant they are to the medication that they are taking and it’s a huge risk to the other patients that are there…then it’s about organising the medication so they can’t just go in and often those facilities don’t have the TB or the ARV treatment. So we need to organise…from our side and make sure the patient either gets…a lot and the doctor needs to write letters as well. It’s not just a simple “I need to go”, there’s a whole medical part that needs to be addressed as well, so the TB patients and obviously MDR and XDR are the people we cannot send and a lot of them are the people with alcohol problems…I can’t expect the patient to go there and wear a mask the whole time that they there…” (Participant 5)

These extracts highlight the importance of collaboration between various service providers, particularly medical staff, when treating substance abusers with infectious diseases such as TB. The logistics of ensuring that adequate medication is available to the patient and is adhered to throughout inpatient substance abuse treatment appears to complicate admissions processes. MDR and XDR TB sufferers are said to be directly prohibited to referral and therefore access to facilities.

### Psychiatric co-morbidity

Referring agents thought that psychiatric co**-**morbidity impeded access to state**-**funded inpatient treatment facilities as disorders needed to be treated sequentially.

“If somebody is psychotic…some inpatient centres even request that if a person has certain mental problems they cannot be admitted to a treatment facility, so when someone is psychotic, or has got a…mental problem…often they can’t go into a treatment facility because mentally they are not stable and that needs to be dealt with before they can be treated in a treatment facility…Once that has been dealt with they can deal with the addiction…however our state- funded facilities are not able to manage them both. If the person is mentally unstable, that needs to be dealt with first before they can work on the addiction.” (Participant 2)

“…if there is a co-morbid psychotic and substance abuse disorder I wouldn’t send them to somewhere like (state-funded treatment centre); they’d first go to (hospital) or (psychiatric hospital) or (psychiatric hospital), one of those places and once they are lucid and their psychosis has subsided then we’d consider sending them to rehab, so it’s always deal with the mental health issue first, if it’s something like psychosis or something really debilitating and then send them to the rehab afterwards, rather than the other way around…” (Participant 5)

“…we do screen the clients…and then we pick up whether, with our psychologists here that you know there is certain mental issues that need to be explored, which we don’t diagnose but then we just refer them on to the psychiatric nurse, she will do a psychiatric evaluation. They will then be medicated, if so, and then after when they are actually stabilised, then we allow them in our treatment facility. But even for those patients that are going through psychosis, we can’t do an application to an inpatient facility, they would never accept that application.” (Participant 4)

Referrals of substance abusers with a co-morbid mental illness were complicated by the fact that they needed to be stabilised before entering state-funded treatment facilities, in which case two referrals were necessary (unless the referring agent was a psychologist). Once stabilised and admitted to the facility, facility staff could continue to treat the co-occurring disorders.

### *Context*-related barriers

This thematic domain attends to barriers to inpatient treatment access situated within Bronfenbrenner’s Context domain that is those domains which pertain to the communities they inhabit and the substance abuse treatment system itself. Referring agents found that general community perceptions were not conducive to the process of accessing inpatient treatment facilities. Cultural beliefs were also found to exert influence on decisions to engage in treatment, the type of treatment, and who could deliver the treatment. Lastly, factors pertaining to the treatment system were also foregrounded for its influence on accessing treatment.

### Lack of awareness

Participants expressed that lack of awareness hindered access to inpatient treatment facilities:

“…people don’t know where to go. These facilities are there but…Our community members don’t know where to go; the only place they will go to is Social Development. So I think that is the big weakness.” (Participant 4)

“Well I think in the (community) community they are quite aware of treatment centres, what is available to them…In other communities—because I’ve worked in the (community) area as well…—people are not that aware…People are not that aware. People living in the area didn’t even know that there was a treatment centre in the area.” (Participant 3)

“There is awareness, but there’s too little treatment centres…so for me the concern is, if they gonna raise awareness and there’s less resources, they still gonna struggle to get in, cause now they know where to get in but I can’t access so why do you tell me…rather look at the resources first and then creating (awareness).” (Participant 1)

Referring agents’ believed that community members are unaware of existing facilities and in instances where they are knowledgeable that treatment was available, exhibited uncertainty about how to access these services. Across communities, levels of awareness differed. It was also cautioned that addressing awareness barriers must occur concurrently with the provision of more treatment centres, or individuals would remain limited in their ability to access treatment services. As referring agents require access points for treatment, individuals need to be aware or need to be made aware of treatment facilities.

### Cultural beliefs

Referring agents’ believed that cultural practices and beliefs in black/African communities hindered treatment**-**seeking:

“…I had a client that…I was about to refer to an inpatient centre, then the family member said no it’s better if we take, like they take the client to Eastern Cape because in Eastern Cape they believe that there isn’t like a lot of drugs…and then some family members would believe that…if maybe they do a ritual for that person, that person would stop using drugs, so there is no need for that particular person to go to an inpatient centre.” (Participant 6)

“…education is important…or maybe like clarifying as to what is being done at an inpatient centre, and then the fact that even if you take a person to Eastern Cape then the person will find drugs there and then clarifying the fact that substance abuse has got nothing to do with rituals, because people believe that okay, this person is misbehaving by using substances because maybe there’s a goat that needs to be slaughtered for that particular person, and then people would believe that the person is going to be okay…” (Participant 6)

Families were more inclined to send the substance abuser to another province or have a ritual performed to address the SUD. Cultural practices also impacted upon who could administer treatment services and how the substance abuser was viewed:

“Our black clients will only be between…18 to 27 beyond that they will never stay in the programme because of language barrier, also because of culture, because they are very set in their way. So if you come in here and you are 45 and you look like at Dada, you look like a Dada but you’re not actually a grandfather, and you see me, I was told once ‘Do you have kids? If you don’t have kids I don’t need your service’. So culture also plays a big role, especially in (community). They relate better to a Xhosa therapist as opposed to any other therapist.” (Participant 4)

“Culturally also men, especially black men, they won’t have an individual therapy session with a female, they won’t; unless you’re married. So all of those little things were picked up, and our stats with regards to retention, with regards to black people, very low.” (Participant 4)

Cultural considerations have a profound influence on the likelihood of access to inpatient treatment facilities in black/African communities.

### Erroneous community beliefs

Erroneous community beliefs also played a critical role in accessing treatment:

“I don’t think they think of it as a disease that needs to be treated…They think it’s the person’s fault; you need to deal with it and to stop it.” (Participant 1)

Referring agents’ believed that community members had little empathy for substance abusers, whom (they believed) were to blame for their substance**-**related difficulties. Moreover, erroneous beliefs about activities at  inpatient treatment facilities show that these services are poorly understood..

“…the perception people have about the inpatient centres, I think it’s not a good one, because they think of that if a person goes to inpatient centres, he or she will come out…like a worse person, like using more drugs and stuff. So I think it’s the mind-set of people, they do not have more information as to what is happening at an inpatient centre…” (Participant 6)

“I think with African people, I think they also in denial about substance abuse they believe that substance abuse is a problem for coloured or white communities they do not really see substance abuse as problem for their communities as well. That’s one of the things-denial about the problem.” (Participant 6)

“And then the other thing that they believe is that when you go to an inpatient centre, drugs will be drained out of their system and then they believe that maybe you will get some injections, painful injections and stuff like that, and so people do not really have like information as to what exactly is happening because I have never heard of any drugs that have been drained out of the system.” (Participant 6)

"Is this how treatment is viewed generally?" (Interviewer)

"No, no, no, when it comes to outpatient centres I think it's because a person wold come for a session and then...go back home, so I think that's the difference because they are seeing that the person has come back unlike when they go to an inpatient centre, the person is going to stay there, and then they think that there are guys who are from (jail), then they are going to get like some information, which is not good information, so when they see a person that goes into an inpatient centre, they only see that the person is going to a place which is the same as going to (jail) whereby the person is gonna get some information about crime and stuff and then the person would have to come back and then be a worse person than he was before." (Participant 6) 

Participants thought that access to inpatient treatment services were limited by community members’ beliefs and poor awareness of treatment services. Poor awareness was also apparent from the belief that substance abuse is a problem for select communities*.* Violence, specifically gang violence, was another barrier identified at the community level.

### Community violence

“…areas like (community) and (community), and not so much (community), the gang violence that takes place is also a big barrier for people to access treatment, like in (community), there is apparently these borders and boundaries within the communities so people from the one side can’t cross over the border to the other side. So if our treatment centre is now on the other side, then they can’t come to use, because they’re placing their lives in danger. (Community) has some similar set-up because of the gangsters that are there. So the whole gang violence, that whole dynamic is there. So that also is a barrier for people accessing treatment.” (Participant 3)

“…it’s quite mind-boggling actually, especially in (community), for me because I was there for a while, how that whole system works, and how you can’t also ask the Police to assist you because then you kind of…the person is going to be seen with the Police van and then that’s going to create another dynamic, and it’s just a whole rigmarole of things, so ja that’s the other thing.” (Participant 3)

Gang violence limited access to referral agents and consequently referrals to inpatient treatment services in disenfranchised communities as service users were unable to reach facilities without compromising their safety and deterring them from doing so.

### Lack of collaboration between service providers

Participants expressed that there is poor networking and collaboration within the substance abuse treatment system. They commented that treatment centres and detoxification facilities worked in silos which was not conducive to service delivery, and by the same token, the process of enacting referrals.

“…there needs to be good networking and interaction between different facilities whether it is: inpatient, outpatient, detox facility. Networking needs to improve…and it needs to be more free-flowing whereby it’s not just about time or…but that it’s a space where things run more smoothly. I think that will help and facilitate the process” (Participant 2)

“I think there isn’t that much collaboration amongst treatment centres.” (Participant 6)

“…networking is also not that great amongst the different service providers. Everybody seems to be doing their own thing, without kind of getting together and having one kind of structure that they follow.” (Participant 3)

### Service providers’ beliefs

Given that referring agents are at the interface of the treatment system and the public, it was also important to explore their views of SUDs. One referring agent appeared to hold stereotypical beliefs of service users based solely on their substance of choice:

“We know that heroin clients aren’t motivated, they’re not, they just want to go into detox that’s it.” (Participant 4)

“And to be honest with you, if not, we came up with this new way forward. We know that with heroin users you can medicate them. We don’t encourage that, we believe in total abstinence with no medication. As long as we don’t make that recommendation. What we do we link the clients and the family up with a physician…of our choice because…he won’t just you know, expect the family to pay money because we know these drugs are very expensive, especially Suboxone, it’s very expensive.” (Participant 4)

This referring agent presupposes that certain service users are less motivated than others, based on their substance of choice. Their personal beliefs also appear to influence practice with regard to their view of substitution medication, though it is conceded that this recommendation is not overtly stated to service users.

### Insufficient treatment facilities

According to our participants, more state-funded inpatient treatment facilities are needed in the Western Cape.

“I definitely think…they (treatment programmes) are effective, but I think the lack of facilities, staff, bed space things like that and the difficulty getting them there is what makes it very frustrating and not that great.” (Participant 5)

“…we’ve got too little treatment centres. We’ve got too little state-funded treatment centres. That is definitely the first thing.” (Participant 3)

“…there is few-especially in other areas-there isn’t any other treatment centres, like for instance in areas such as (township), (township), (township), there isn’t even one treatment centre, so I think those are some of the weaknesses that there is like few, that they still need like, they still need to kind of have more treatment centres in other areas as well.” (Participant 6)

Our respondents emphasised that more state**-**funded treatment facilities, which are evenly dispersed, are critical for improving service delivery. Related to the limited capacity of the treatment system, service users were often subject to protracted waiting time for admission to treatment.

### Waiting time

According to all referring agents, protracted waiting time before admission to treatment facilities was commonplace.

“The other weakness would be moving from an outpatient to an inpatient; the waiting period is just way too long. If you want to refer someone to inpatient, and this I speak from experience, the waiting period is anything from 6 to 9 months…it’s a great difficulty for us, because we are even to scared to offer the client inpatient when they come here, because we know what the waiting period is going to be like and it’s very difficult to keep people motivated for so long…” (Participant 3)

“Maximum time period for a bed, for admittance would be then three until six months, and that is just one of the barriers. The process alone is tedious, people don’t have the patience.” (Participant 4)

“…you know with the addict he uses everything and anything as an excuse. So if he doesn’t get in when he wants to get in, eventually it’s gonna be…it’s because…the treatment facility wasn’t able to take me. It’s because I had to wait three months and that is why I’m back at using and I didn’t stop using so…we just become pulled into that where they use us and they blame us…” (Participant 2)

For some service users, waiting time decreased their motivation for treatment and became the rationale for continued substance use. Waiting periods were reported to be so lengthy that one referring agent conceded that she was reluctant to suggest a referral. Another participant reflected that there were waiting periods at every stage of the inpatient application.

“So barriers is waiting time for that application to be processed, assessed at the inpatient facility. Another barrier would be the waiting period for admission that is too tedious. Another bad area is the fact that if it has to go through statutorial lines it’s much more longer…” (Participant 4)

Where detoxification services were needed, waiting lists were also reported.

“…if you are a heroin user we will apply for inpatient for that person. We already were supposed to have completed that application having a date at hand already before (psychiatric hospital) will basically admit you. That is more than seven months that you’re going to wait honestly…” (Participant 4)

Extensive waiting time for enacting referrals and eventual access to the treatment facility was emphasised by study participants and said to last between 3 and 9 months. While decreased motivation for treatment was one way in which eventual admission to the inpatient treatment facilities became less likely, other consequences of waiting included becoming psychotic, being imprisoned, or dying.

“…addiction: it’s a progressive illness—not only does the client use more but the damages in their life becomes more if they don’t have, if they don’t access help, and I mean if they don’t access help then they continue using and it could mean life or death, it could mean becoming psychotic or not, it could mean landing up in jail or not, you know, even dying. We do say that the end result of using is death, mental institution or jail. So if he doesn’t get the help when he really desperately wants it, and we procrastinate and we wait, and we struggle to get him the help, in the meantime we know he can die…” (Participant 2)

### Referrals to state-funded inpatient treatment facilities

#### Administration

Participants expressed concern about the administration at state**-**funded inpatient treatment facilities.

“I also think their administration needs to be reviewed. I think the point of entry to the exit is questionable.” (Participant 4)

“I’ve been trying to refer one patient since December last year, she’s still not in, and it’s just the typical example that I will phone and say “what’s going on?” No we need this form, then they will send the form…then we send it off, then I wait, then a month later I go “what’s going on?” “No, you weren’t meant to send the form, the social worker was meant to”, I said “but… why didn’t you phone me and tell me?” so there’s absolutely no communication-nothing! They don’t phone to say your form is incomplete or…“actually, you can’t send the form” and when they do say they’ll phone you back…I’m still waiting for the phone call from two weeks ago.” (Participant 5)

“Lack of follow up on their part, if a form is incomplete or they need something else then they just—unless you phone them back and ask, and they go and check-you won’t hear from them.” (Participant 5)

Practices at state**-**funded inpatient treatment facilities were viewed as hindering access to these services. Various administrative processes were questioned, as was the failure of these facilities to communicate administrative needs, which impacted upon the success of referrals.

### Social worker reports

According to referring agents, referral applications must be compiled by a social worker.

“…the social workers in a way, they play an important role in terms of helping the people to access inpatient treatment.” (Participant 6)

“With the government funded facilities, they require a referral mostly from either an outpatient facility or it’s done by social workers…” (Participant 3)

Clinical psychologists were said to be unable to make referrals, as it was in the purview of social workers to compile the referral documentation.

“…they are not very keen on me making referrals. They want-and I’m speaking specifically about (state-funded inpatient facility) that I’ve had huge issues with…—they want a Social Worker to refer them, and…when I’ve asked this in the past, you know “why” or “why can’t I do it?” they say because they want to refer the patient they have to after discharge refer the person back to the social worker, whoever in the community or whatever…so that’s how I understand the actual procedure that we, I pick it up, I need to then refer to whatever other social development or social worker in our clinic, and once they have done an assessment then the social worker writes the referral letter and then sends them off to…or they go on the waiting list and then they phone and they go in hopefully.”

“…I’ve said to them I don’t understand why I cannot do the referral, I’m more qualified than a social worker and they still would say ‘oh but we need a social worker’…I don’t think they really understand-the people on the end of doing the admissions-I don’t think they actually understand what my title is and my role and how it differs to a social worker, that we also dealing with these sorts of issues, I don’t think they get that and I think they just have on paper that they must have the social worker, so they’re stuck in that mind-set.” (Participant 5)

Unable to make direct referrals, this psychologist partnered with a social worker to refer patients. Challenges such as conflicting work schedules delayed referrals, as messages were passed along through other staff.

“…usually I incorporate the social worker, get her to do an assessment… and together I try and make-because of me being told that the social worker needs to be involved-then I would do the referral with that…but because the social worker and myself are probably most of the time not here on the same day it creates a huge problem because there’s a lack of communication between-just because of logistics between us as well—because she’s not here so I can’t walk to her office and say “okay, this and this please follow up from the patient”, so it’s literally, again, passing messages through other people, you know, once I leave that clinic, the next day I’m at another one and it comes with its own issues so I can’t-it’s so difficult to keep on following up on past stuff at every clinic, I don’t have time really…usually just getting the social worker involved and trying to do it together and then the social worker normally has the forms and…would fill them in…and then fax it off and then it’s sort of a waiting game…” (Participant 5)

In contrast to the difficulties reported by the psychologist, another participant, a registered counsellor stated that she had no difficulty in referring service users:

“Okay, so we’ve got a working relationship with our inpatient facilities, so I would have a relationship with somebody at that inpatient. I’d contact them and tell them look I’ve got a client…I need to get him in, his not coping in our outpatient facility. That person would obviously tell me fill out the referral form. I’ll sit with the client, fill out the referral form and then send it off to the inpatient, and then they’ll obviously let me know when they have space available.” (Participant 3)

This however suggests that practices are not uniform throughout the referral system.

#### Referral documentation

Participants remarked that it was challenging and time-consuming to complete referral forms.

“I must say that the referrals are also not that easy.” (Participant 3)

“…the paperwork is too long…” (Participant 3)

“For the one inpatient facility the referral form is basically an assessment, so in that you would cover the person’s medical history, their…childhood, about their family, their support structures…their psychiatric history…information about their substance use history…any life-changing events that could have maybe contributed to the person actually starting to use… some of the inpatient referral…they not one form, it’s like a little pack. It’s like doing an assessment of the client and sending that off …” (Participant 3)

“…the one inpatient facility’s referral form…it’s so thick that you actually take, you actually take about an hour and a half with your client to fill it out... if you’ve done your assessment you have most of the information that you can fill out, but you have to sit with the client, there’s additional stuff that they want and you have to sit and fill it out …” (Participant 3)

“So now it’s also a lengthy process, and that’s gonna determine with…the admin of the facility, if it’s a lot of paperwork, if they require a lot of things from you, like the salary slip, and an affidavit, and you know, lot of things…” (Participant 1)

“…to go to inpatient there’s all of that legislature and process involved in actually making that happen.” (Participant 4)

“So it’s just red tape, which really it doesn’t make sense because even though the client comes today and I can see the severity of his withdrawal symptoms, application might take three days for me with my psycho-social report. That is sent onto inpatient. When they phone us it might take four months for the most, three to four months. So it means that that person can’t detox. So it doesn’t make sense.” (Participant 4)

These extracts, however, also suggest that referral agents have a limited understanding of their role.

Participants noted further barriers to treatment directly related to enacting referrals:

“There’s lots of barriers…some of them lose your admin, and you have to do it over again, though you know, and that, when the admin is sort of lost somewhere in transit then you as the referee look unprofessional because the client is depending on you to get things in order to you.” (Participant 1)

The extract below conveys this referring agent's frustration at their inability to expedite referral processes.

"It's very time-consuming...even that paperwork is really time-consuming...it's quite intensive...you really need time out to actually sit and do it, and then obviously the follow- up process; to give them a courtesy call like 'when do you think you'll have space?'- you know that type of thing. It's quite a drawn out process and it becomes quite demoralising sometimes, because you just don't even want to go through that." (Participant 3)

Referring agents who held professional titles other than those of a social worker perceived the requirement of a social work report as an additional barrier to accessing inpatient treatment as they were obliged to work with social workers, which lengthened the time taken to complete a referral. Completed referral documentation also did not guarantee that the application process was complete as some facilities were reported to lose paperwork, which had to be compiled again. The referral process was described as time-consuming, labour-intensive, and at times, “demoralising”. Referring agents also exhibited little knowledge of their role. The referral system, the very means by which access to inpatient treatment facilities is facilitated, appears to act as a barrier.

### Homelessness

Homelessness was believed to impede access to state**-**funded inpatient treatment facilities:

“…if somebody doesn’t have a physical address where they go back to, then they cannot be admitted to a treatment facility…they need to have an address that they need to go out to….often we have to negotiate and try and get the person into a shelter and use the shelter’s address as a fixed abode…really taking that step to getting help still while you are on the street is already a big step that you taking and to be shown away because you don’t have a place to stay is a concern, but unfortunately that is our system.” (Participant 2)

“…homelessness because the treatment system won’t take the person if they don’t have an…address and if they’re being discharged, they want that, so that is the difficulty.” (Participant 1)

Treatment system eligibility criteria, that is, inpatient treatment centres’ requirement of a residential address on referral documents, limited access to treatment for the homeless.

### Uninformed treatment staff

Staff within the substance abuse treatment system were described as being uninformed, and consequently, missed windows of opportunity to assist service users by suggesting and facilitating referrals.

“The other weakness is the fact that staff, manpower; people aren’t educated within the substance abuse field, as experts. I think in this field you do really need to have some form of expertise, not everyone can do substance abuse therapy. No, not everyone.” (Participant 4)

“I think if they train the social workers, and if facilities come to clinics, and just spend ten minutes in one of our meetings and explain how it works, can a doctor refer, can a nurse refer, what do you do?, that would be really helpful because they don’t know, they really don’t know. Often I’ll go to different nurses or different meetings, different clinics to explain my process and substance abuse always comes up, what must we do with these people? They really don’t know.” (Participant 5)

Participants also described geographical barriers that were faced by service users attempting to access substance abuse treatment services.

### Geographical barriers

“People can’t access our treatment centre because transport is a problem, they don’t have money, they can’t travel so…that is a big barrier.” (Participant 3)

“…they don’t have money for transportation, to be honest some of our clients walk from (suburb), even from (suburb), that’s how committed they are. But you can’t expect everyone to have the same level of commitment…transportation is our big, big concern.” (Participant 4)

“…they actually have to walk till here and it’s not very safe for them to walk…Safety. The transport routes are not always ideal for where the centres are located, so I would say we need more outpatient facilities in the broader community.” (Participant 3)

The locations of existing outpatient treatment facilities were said to limit access to referral agents as they are not centrally located and are not conducive to travelling to by public transportation. Service users from various communities undertook the long journey to outpatient facilities or walked far distances when no money for transportation is available, often compromising their safety.

### Financial barriers

Limited finances compelled many service users to rely on state-funded treatment services. Although these services are free, travel costs were still incurred when having to meet with referring agents at outpatient centres and when referrals were successful when travelling to inpatient treatment centres. Often treatment slots could not be occupied due to other priorities within the home.

“…they struggle with getting here, because a lot of the time we deal with people that…don’t have money they find it difficult to travel…” (Participant 2)

“More often than not, with the client that we see in our treatment centre, in their household there’d be no breadwinner. So they would be living off either a (child support) grant, or the parents will be getting a pension or a disability grant which is so little money…they still have to kind of find means of getting to a treatment centre, they do not have the money to travel…even if they can get to a state—funded outpatient facility like ours they don’t always have the money to get to us…so that also becomes a barrier, or that keeps people from seeking treatment. They might come the one day because they managed to get money to come, but how are they going to maintain it for another 6 weeks?” (Participant 3)

“it’s simple things like the patient getting to the facility, some of them literally don’t have a cent, so just for them to get from our facility whether they have to go for an outpatient, an interview or whatever it might be, to get there is a barrier…” (Participant 5)

These extracts convey that finances play a critical role in attempts to access outpatient and inpatient treatment.

## Discussion

The major findings of this study are that service users encounter numerous access barriers to state-funded inpatient substance abuse treatment. Barriers challenge efforts to reduce alcohol and other drug harms at the individual, community and societal level by limiting successful linkage to care, and therefore, individuals’ capacity to participate in and ultimately benefit from treatment. Identified barriers were primarily external to the individual, related to the substance abuse and referral system, and in the presence of service users having specific characteristics (e.g. being homeless, pregnant or having a disability), limiting access to treatment for specific subgroups. The success of referrals, the prerequisite for receiving treatment at state-funded inpatient facilities, were impacted upon by lengthy application forms, administrative processes at the receiving facility, lack of communication and collaboration amongst various links in the referral network, deficits in practitioner knowledge and limits on who was allowed to make referrals. Participants verbalised that community dynamics impacted upon access to treatment and that at a most fundamental level, these were driven by community members’ lack of understanding about addiction as a disease, and consequently, an inability to identify that there was a need for a behavioural intervention. Erroneous beliefs were also held about activities at treatment centres, although to lesser extent when it came to outpatient treatment. Community violence, specifically gang activity, limited initial contact with treatment services and referring agents, and in so doing, to inpatient treatment facilities. Specific barriers identified for female service users included added stigma, and where this was overcome, system barriers constrained treatment options for pregnant service users. Unmet childcare needs often determined if women could take up treatment slots.

In contrast to past research where internal barriers were central in hindering treatment access (e.g. [12]), the vast majority of barriers identified in this study were external to service users. System factors which manifest as fragmented service delivery signal the importance of our research for harm reduction in our context, and our recommendations are outlined in the concluding section of this paper.

Earlier research confirms the importance of motivation in decisions to access treatment (see [12]), and as noted by one participant, contact time with treatment users should be used to enhance their motivation. With reference to the barriers identified for women, international research has noted that women are more likely to be stigmatised [16] and be ineligible for entry to treatment when pregnant [17]. Related to our findings that unmet childcare needs lead women to forego opportunities for treatment, it has been demonstrated that when programmes meet service users’ needs for childcare, access is optimised see [18].

International evidence also shows that service users with disabilities are underrepresented in treatment due to access and other barriers [19], but it is interesting to note that in this study this is attributed to the physical characteristics of treatment facilities and the medium used to deliver the programme, suggesting an element of discrimination (see [19]). Tuberculosis was found to complicate and in some instances prohibit referrals to inpatient substance abuse treatment, with part of the admissions process including obtaining adequate medication to be used for the duration of their stay at the facility. TB must be ruled out diagnostically amongst suspected sufferers or be treated prior to admission to substance abuse treatment facilities, with access to treatment being prohibited in the interim [20].

Referral agents’ concur with a South African access study on the barriers to inpatient and outpatient treatment [2], namely the absence of a structured referral pathway, the need for formal referrals from a social worker, long referral processes, having to undergo mandatory detoxification or mental health treatment services prior to admissions to treatment facilities as these services are not integrated, service user eligibility criterion and extensive waiting time.

Former research notes the lack of intersectoral collaboration between government departments [11], whereas our participants foregrounded the lack of collaboration between service providers as leading to fragmented service delivery. With respect to the barriers faced by black/African South Africans, our results differ from those of earlier work ([2], p. 100–101) which found that “rather than seeking treatment in the early stages of the illness, (black/African) communities tend to seek treatment only once the problem has become severe…as a result, communities tend to demand access to inpatient treatment and are dissatisfied when treatment slots are not immediately available.” Driven by a lack of understanding of practices at inpatient substance abuse treatment centres, service users preferred outpatient treatment services, or, in other families, the substance abuser was sent to another province or had a ritual performed on their behalf.

Waiting time for treatment and the deleterious effects thereof for access to treatment noted in this study are well-documented in earlier studies within South Africa and beyond [11, 21].

Research in our context has also shown the location of treatment facilities on its own and in conjunction with financial barriers related to commuting to treatment services to hinder access to treatment for service users in disenfranchised communities who need to ensure that their basic survival needs are met [22].

This study’s qualitative methodological framework limits the generalizability of its findings; however, results may be transferable to other contexts [23]. Only one referring agent worked within a black/African community setting, which limited the researchers’ ability to explore all the nuances in barriers faced in these settings. The study would also have been strengthened if service users and service providers had been interviewed, and this provides some direction for future research.

## Conclusions

The challenge of access barriers to inpatient treatment facilities is salient, and our findings have implications for harm reduction in the Western Cape. A more enabling environment can be fostered through a focus on infrastructure, policy and the role players within the treatment system. Regarding infrastructure, we recommend that outpatient and inpatient treatment facilities must be made more inclusive of the needs of persons with disabilities, and this may lead to changes in the characteristics of treatment facilities such as the provision of ramps, for example. With respect to policy, eligibility criteria, such as the need for a physical address to be listed on referral documents, must be reconsidered. Referrals can be simplified by standardising referral forms, which also need to be more succinct. Community engagement led by trained outreach workers can be used to challenge negative attitudes and beliefs about substance users and treatment. Lastly, as concerns were raised about the workforce rendering treatment and referral services, these individuals need to be educated as to their role and be incentivised to improve their professional competencies. In recent years, a postgraduate diploma in addictions care has been developed to improve the skills of the treatment workforce, and it is imperative that more practitioners receive the training. It is hoped that the South African government’s efforts to increase the number of treatment facilities will positively impact upon waiting time and financial barriers that were noted in this study.

## Endnote

The terms “coloured,” “black” and “Indian” were racial categories used during South Africa’s apartheid era to reinforce a segregated society and to refer to those who were not afforded the same benefits as whites. The terms are used here merely for descriptive purposes and do not imply our acceptance thereof.

## Appendix A: interview schedule

### Substance abuse treatment system

1. How does the substance abuse treatment system operate in the Western Cape?

2. In your opinion, what are the strengths of this treatment system?

3. What are the weaknesses of this treatment system?

4. Within your context, do you consider the substance abuse treatment system to be effective? Please elaborate.

### Procedure for gaining entry into inpatient treatment facilities

5. If a person were interested in seeking treatment for their substance use disorder, how would they go about doing so? Could you briefly outline this step**-**by**-**step process?

6. How are individuals with your job title/job description instrumental in this process?

### Barriers to substance abuse treatment entry

7. In your experience, are there any factors or events that serve as barriers to entering into inpatient substance abuse treatment facilities?

8. Are these factors characteristics inherent of the treatment system or individuals?

9. Are there any individuals you do not refer to treatment? Why is that?

10. Typically, which individuals or subgroups come here in need of help?

### Enabling factors to treatment entry

11. Are you able to identify any factors that ease the process of entering into inpatient substance abuse treatment centres? Are these factors related to individuals or the treatment system?

12. Do you perceive that all individuals in need of inpatient substance abuse treatment are ultimately able to access the aid they need? Please elaborate.

13. What would you suggest be changed or put in place to make accessing inpatient treatment easier?

### Service provider

14. In your opinion/experience, is substance abuse a treatable illness? Could you briefly elaborate?

15. Is there anything you would like to add that I may have neglected to ask about accessing inpatient treatment?
